# Biochemical and structural insights into the cytochrome P450 reductase from *Candida tropicalis*

**DOI:** 10.1038/s41598-019-56516-6

**Published:** 2019-12-27

**Authors:** Ana C. Ebrecht, Naadia van der Bergh, Susan T. L. Harrison, Martha S. Smit, B. Trevor Sewell, Diederik J. Opperman

**Affiliations:** 1 Department of Microbial, Biochemical, and Food Biotechnology, University of the Free State, Bloemfontein, 9301 South Africa; 2 Centre for Bioprocess Engineering Research (CeBER), Department of Chemical Engineering, University of Cape Town, Rondebosch, Cape Town, 7701 South Africa; 3 South African DST-NRF Centre of Excellence in Catalysis (c*Change), University of Cape Town, Private Bag, Rondebosch, Cape Town, 7701 South Africa; 4 Structural Biology Research Unit, Department of Integrative Biomedical Sciences, Institute for Infectious Diseases and Molecular Medicine, University of Cape Town, Cape Town, 7700 South Africa

**Keywords:** X-ray crystallography, Oxidoreductases, Biochemistry, Biotechnology

## Abstract

Cytochrome P450 reductases (CPRs) are diflavin oxidoreductases that supply electrons to type II cytochrome P450 monooxygenases (CYPs). In addition, it can also reduce other proteins and molecules, including cytochrome *c*, ferricyanide, and different drugs. Although various CPRs have been functionally and structurally characterized, the overall mechanism and its interaction with different redox acceptors remain elusive. One of the main problems regarding electron transfer between CPRs and CYPs is the so-called “uncoupling”, whereby NAD(P)H derived electrons are lost due to the reduced intermediates’ (FAD and FMN of CPR) interaction with molecular oxygen. Additionally, the decay of the iron-oxygen complex of the CYP can also contribute to loss of reducing equivalents during an unproductive reaction cycle. This phenomenon generates reactive oxygen species (ROS), leading to an inefficient reaction. Here, we present the study of the CPR from C*andida tropicalis* (*Ct*CPR) lacking the hydrophobic *N*-terminal part (Δ2–22). The enzyme supports the reduction of cytochrome *c* and ferricyanide, with an estimated 30% uncoupling during the reactions with cytochrome *c*. The ROS produced was not influenced by different physicochemical conditions (ionic strength, pH, temperature). The X-ray structures of the enzyme were solved with and without its cofactor, NADPH. Both *Ct*CPR structures exhibited the closed conformation. Comparison with the different solved structures revealed an intricate ionic network responsible for the regulation of the open/closed movement of *Ct*CPR.

## Introduction

Cytochrome P450 reductases (CPRs or CYPORs, EC 1.6.2.4) are diflavin oxidoreductases that supply electrons to type II cytochrome P450 monooxygenases (CYPs, EC 1.14.-.-)^1^. *In vivo*, CPRs also reduce other proteins such as cytochrome *b*_5_, heme oxygenase, and the fatty acid elongation system; *in vitro*, it has been shown to transfer electrons to non-physiological acceptors such as cytochrome *c*, ferricyanide, and different drugs^2^.

CPRs are present in most eukaryotes, and a few prokaryotes where it is fused to a CYP domain as is the case with CYP102A1 (P450BM3)^3^. The structure is well conserved among the different kingdoms, possessing an *N*-terminal FMN-binding domain that is connected to a *C*-terminal FAD-binding domain via a linker region. In most CPRs, the *N*-terminus of the protein (~6 kDa) acts as a membrane anchor and is responsible for its localization in the endoplasmic reticulum^4^. The enzyme transfers electrons from NADPH in a typical order via flavin adenine dinucleotide (FAD) to flavin mononucleotide (FMN), which ultimately shuttles the electrons to the protein acceptors. The proposed mechanism is that during inter-flavin electron transfer, the enzyme is in a compact, closed conformation which facilitates the electron flow between the prosthetic groups. However, this conformation is not ideal for interaction with protein acceptors and therefore CPR undergoes conformational changes to expose the reduced FMN for interaction with CYPs^5^.

Different techniques have been used to study the domain movements and CPR-acceptor interaction: X-ray crystallography^4,6,7^ NMR^8–10^ small-angle X-ray scattering (SAXS)^9,11^ small-angle neutron scattering (SANS)^8,11–13^ fluorescence resonance energy transfer (FRET)^14,15^ neutron reflectometry (NR)^16^; electron double resonance^17^; and mass spectrometry^18^. Despite the different approaches, the overall mechanism and details of the CPR interaction with its redox acceptors remain elusive and further analysis is necessary to completely understand the dynamics of electron shuttle to the CYPs.

One of the main problems regarding electron transfer between CPRs and CYPs is the so-called uncoupling phenomenon, which generates reactive oxygen species (ROS). ROS result in loss of electrons and leads to an inefficient reaction, oxidative damage and ultimately inactivates the enzymes^19^.

*Candida tropicalis* possesses two CPRs, functioning in the transfer of electrons to its CYPome, including CYP51 (sterol 14α-demethylase) and, most notably, a variety of CYP52 (fatty acid and alkane hydroxylases)^20^. In this study, we investigated ROS production by *C. tropicalis* CPR (*Ct*CPR) under different conditions. We produced a truncated (Δ2–22) version of the enzyme (Fig. S1) that supports not only the reduction of cytochrome *c* and ferricyanide, but also CYP52A21 from *C. albicans*. We analysed the production of H_2_O_2_ and superoxide during *Ct*CPR activity, as well as the influence of different physicochemical parameters on uncoupling. In addition, we solved the structure of *Ct*CPR with and without the cofactor, NADPH. Both structures revealed *Ct*CPR in the closed conformation. The overall structures are similar to those previously reported for mammalian and yeast proteins^21–23^. We discuss the differences and similarities between the different CPRs and review the regulatory function of inter-domain ionic interactions.

## Results and Discussion

### Protein expression and spectral properties

**Figure 1:**
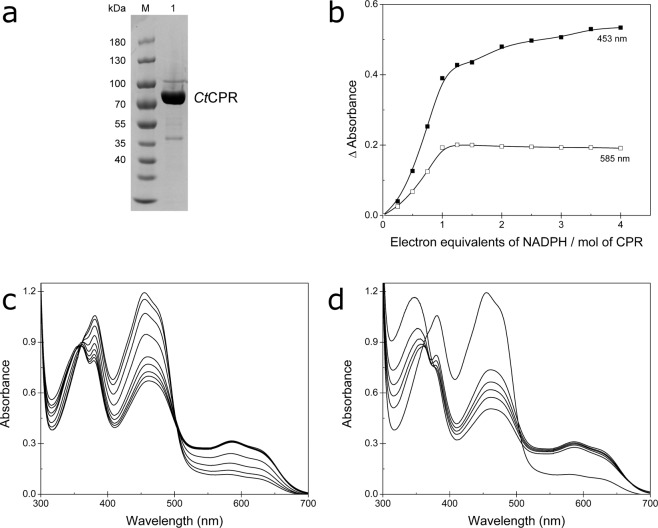
(**a**) SDS-PAGE of purified *Ct*CPR (lane M: molecular weight marker, lane 1: *Ct*CPR after three-step purification). (**b**) Changes in absorbance at 453 nm and 585 nm peaks after addition of different equivalents of the cofactor. (**c**) Spectral properties of *Ct*CPR. Changes in absorbance were recorded after the addition of NADPH (up to four equivalents per mol of CPR). (**d**) Spectral change after addition of excess NADPH (more than four electron equivalents per mol of CPR).

*C. tropicalis* possesses two genes coding for CPR, *cpr-a* and *cpr-b*^24^. The genes are alleles with high identity between the protein sequences and have similar activity rates. In this work, we expressed the *Ct*CPR coded by *cpr-b*. The *N*-terminal domain of eukaryotic CPR is a highly hydrophobic sequence that acts as a membrane anchor. One strategy for the solubilization of membrane-associated proteins is the deletion of this hydrophobic region, which generally improves heterologous expression in *Escherichia coli*^25^. Analysis of the *Ct*CPR sequence revealed the presence of a single membrane-spanning region comprising the first 22 amino acids of the protein and in an attempt to produce more soluble CPR, a truncated, untagged version (Δ2–22) of the enzyme was expressed in *E. coli*. Protein purification was a three-step procedure involving low- and high-resolution anion-exchange chromatography, followed by size-exclusion chromatography, to achieve a high level of purity (Figs. 1a and S11).

The UV-visible analysis showed the typical spectrum for CPRs, with absorbance maxima at 380 and 453 nm and a shoulder at 480 nm, which are indicative of oxidized FAD and FMN. Based on the average concentration of these peaks, we estimated a yield of 0.2 mmol of *Ct*CPR per liter of *E. coli* culture (340 mg/L). For titration of the flavin groups, increasing concentration of NADPH was added and absorbance spectra were recorded after reaching equilibrium (Fig. 1c). Titration with NADPH under aerobic conditions showed similar results to other CPRs^5,7,26–31^. Low concentrations of NADPH (less than one electron equivalent per mol of CPR) led to a decrease in the 453 nm peak and the appearance of a broad peak with maximum absorbance at 585 nm and a shoulder at 630 nm (Fig. 1b,c), indicative of the formation of the air-stable semiquinone^28^. Further addition of NADPH produced little change in the semiquinone peak and a decrease in absorbance at lower wavelengths, which represents the transition into different states of the flavin groups. Higher concentrations of NADPH (more than four electron equivalents per mol of enzyme) resulted in accumulation of the cofactor (increase in absorbance at 340 nm) and slight variations in the enzyme spectrum (Fig. 1d). It is well established that the directional flow of electrons during CPR catalysis is initiated by a hydride transfer from NADPH to the FAD group, where after direct inter-flavin electron transfer takes place. Finally, the electrons are shuttled to the external acceptor from the FMN group. However, no consensus has been reached concerning the reducing cycle and the active form of FMN that donates electrons to the acceptor. It has been shown that mammalian and plant CPRs can be completely reduced (4 electrons) with excess NADPH^15,31,32^, but *in vivo* the enzyme most likely cycles between the 1- and 3-electron reduced states, where the FAD-FMNH· semiquinone is the resting state and the electrons are delivered to the acceptor by the hydroquinone form (FMNH_2_) during catalysis^2,27,28^. In contrast, for other CPRs including the CPR-domain of P450BM3, a reduction cycle of 0-2-1-0 has been described, where the resting state of the enzyme is the fully oxidized form and the semiquinone FMNH· transfers the electrons^23,33–36^.

### *Ct*CPR activity and kinetic analysis

Despite *N*-terminal truncation, *Ct*CPR was fully active and able to reduce both cytochrome *c* and FeCN. The rate of conversion for cytochrome *c* was in the same order as for FeCN (*k*_cat_ 7480 min^−1^ and 9398 min^−1^, respectively). Saturation curves for both substrates followed Michaelis-Menten behaviour (Fig. S2), with a *K*_M_ of 360 µM for FeCN and 4.5 µM for cytochrome *c* (Table 1). Although these molecules are not physiological substrates of the enzyme, they are commonly used to evaluate CPR activity due to the simplicity of the assays. FeCN, and other small molecules, only require one electron, which can be donated directly from FAD^10^. Cytochrome *c* reduction is proposed as an analogue to natural substrates^37^, but being a smaller protein, it does not share the same reduction mechanism or binding site as the CYPs^38,39^. More recently, it has been proposed that cytochrome *c* can receive electrons without binding to the reductase^40^. Nevertheless, cytochrome *c* is often used for CPR characterization and *Ct*CPR was previously purified from *C. tropicalis* and characterized as an NADPH-cytochrome c reductase^41^. The catalytic efficiency of *Ct*CPR appears to be greater (at least 10 times) than for most other CPRs, with the exception of *S. cerevisiae* CPR, which exhibits a 2-fold higher *k*_cat_/*K*_M_ (Table 1).

**Table 1:** Kinetic parameters for cytochrome *c* reduction by CPR from different species. n.d., not determined.

Organism	*K*_M_ (μM)	*k*_cat_ (min^−1^)	*k*_cat_/*K*_M_
*Candida tropicalis* (recombinant)^this work^	4.5	7480	1662
*C. tropicalis* (native)^41^	4.3	6100	1418
*C. apicola*^56^	13.8	1915	139
*C. albicans*^63^	n.d	2830	—
*Saccharomyces cerevisiae*^53^	2.4	8940	3725
Rat^64,65^	21.1	3000	142
Human^53^	3.1	1800	580
*Musca domestica*^33,34^	4.6	3024	657
*Chilo suppressalis*^66^	14.3	1117	78
*Gossypium hirsutum* ATR1^67^	1.2	239	199
ATR2	1.6	605	378
*Andrographis paniculata* CPR1^68^	73.15	335	4.6
CPR2	12.89	73	5.7
*Capsicum annuum*^69^	81	2740	34
*Botryococcus braunii*^70^	11.7	198	16.9
*Bacillus megaterium*^29^	16.7	2582	154

As some N-terminally truncated and heterologously expressed CPRs have been found to support the reduction of artificial electron acceptors but not CYPs^42^, we tested the ability of *Ct*CPR to support the reduction of the previously characterized CYP52A21 from *C. albicans*^43^. GC-MS analysis of the biotransformations with dodecanoic acid showed the expected production of 12-hydroxydodecanoic acid (Fig. S3), confirming *Ct*CPR’s capability to support CYP activity.

### Uncoupling under different physicochemical conditions

The CPR supplies electrons to a wide variety of microsomal CYPs that are involved in important metabolic functions *in vivo* and are highly valuable for medical and technological applications^44^. One of the main problems of the CPR-CYP system, however, is the instability and low activity of the enzymes. During electron transfer, uncoupling can produce ROS species through the non-productive activation of oxygen, generating H_2_O_2_ and superoxide anions^45^. The mechanism and factors that influence this phenomenon are not completely understood, but it is known that the coupling efficiency varies depending on the P450 system used^19^, as both CYP and CPR can contribute to ROS formation. Most studies have focused on ROS production by CYPs, but it has been shown that CPR can accept electrons from NADPH in the absence of an acceptor, generating H_2_O_2_ and superoxide radicals^19,46,47^. To investigate the contribution of *Ct*CPR to uncoupling, we measured the consumption of NADPH in the presence and absence of cytochrome *c* in parallel with the formation of ROS. We used a previously described spectrophotometric method to quantify the production of both H_2_O_2_ and superoxide, by the addition of superoxide dismutase^19^. During the assays, we noticed that *Ct*CPR was consuming more cofactor than the quantified H_2_O_2_ formed. Different controls showed that in the presence of Ampiflu Red (10-acetyl-3,7-dihydroxyphenoxazine) and/or HRP the enzyme used more NADPH than in the absence of these components (Fig. S4), indicating that *Ct*CPR could be interacting with the Ampiflu Red and HRP. To overcome this issue, we separated the reactions and first measured NADPH consumption (or NADPH consumption and reduction of cytochrome *c*) and after enzyme inactivation quantify the ROS generated. Relevant controls confirmed that the heating of the samples did not affect ROS production (Fig. S5).

**Figure 2:**
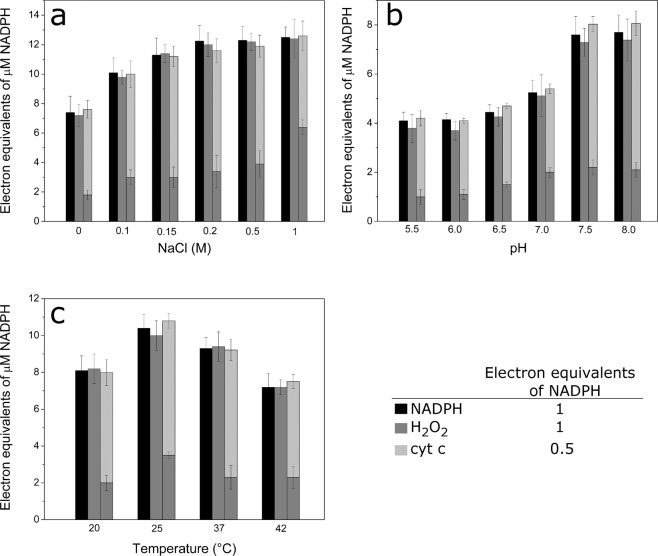
Uncoupling of *Ct*CPR under different conditions. Bars represent NADPH consumed (black), H_2_O_2_ produced (grey) and cytochrome *c* reduced (light grey). (**a**) Effect of ionic strength on the uncoupling was performed at different concentrations of NaCl (0–1 M). (**b**) The effect of pH was analysed using 30 mM sodium phosphate buffer (pH 5.5–8.0). (**c**) To analyse the effect of temperature on uncoupling, reactions were carried out at different temperatures (20–42 °C).

In the absence of cytochrome *c, Ct*CPR consumed NADPH and produced equivalent amounts of ROS. H_2_O_2_ was the major product of uncoupling, since the addition of SOD only slightly increased the total amount of H_2_O_2_ detected (Fig. S6). Similar results were obtained for *C. apicola* CPR^19^. We observed similar levels of NADPH consumption in reactions with cytochrome *c*, although the electrons were mainly used to reduce the acceptor and ROS production was on average 3 times lower compared with reactions without cytochrome *c* (Fig. 2). We estimated an uncoupling of ~30% (Table S2). Since it has previously been reported that different factors can affect the coupling efficiency of the system^45^, we investigated the effect of different physicochemical conditions on *Ct*CPR activity and their influence on ROS production.

We analysed the effect of ionic strength by performing the reactions with different concentrations of NaCl. NADPH consumption rates increased with ionic strength up to 1.7-fold compared with reactions with no salt. No significant differences in NADPH consumption were observed above 0.2 M NaCl (Fig. 2a). For the reactions with cytochrome *c*, the activity of *Ct*CPR was also higher in presence of salt but an increase in ROS production was also observed, with the total uncoupling remaining close to 30% (Table S2). Previous work^9,11,48^ has shown that higher ionic strength favours the open conformation of the CPR, increasing the catalytic efficiency towards cytochrome *c*. Only at the highest ionic strength tested (1 M) the uncoupling increased to 50%. SANS data showed a considerable increase in the flexibility of the enzyme, suggesting that the CPR might be partially unfolded under high ionic strength^13^.

In addition, we examined the impact of pH and temperature on uncoupling. It has been shown that *Ct*CPR’s optimal pH is between 7.5 and 8.0 and that the enzyme exhibits a decrease in the activity at lower pH values^41^. This is in agreement with the results obtained in this work, where NADPH utilization was greater at more alkaline pH and the reduction of cytochrome *c* was ~50% less under neutral and acidic conditions (Fig. 2b). SAXS analysis of human CPR at two different pH values showed a shift in the closed-open equilibrium: the closed population increased at lower pH (6.7), while the open conformation seems to dominate at higher pH (7.4)^9^. These findings correspond with the idea that an open state favours the activity of the enzyme. Interestingly, the percentage of uncoupling was not influenced by different conditions (Table S3).

*Ct*CPR exhibited optimal activity at 25 °C (Fig. 2c), but a similar percentage of uncoupling compared with the other temperatures, with only a slight reduction (5%) at 37 °C (Table S4).

### Crystal structure of the *Ct*CPR

**Figure 3:**
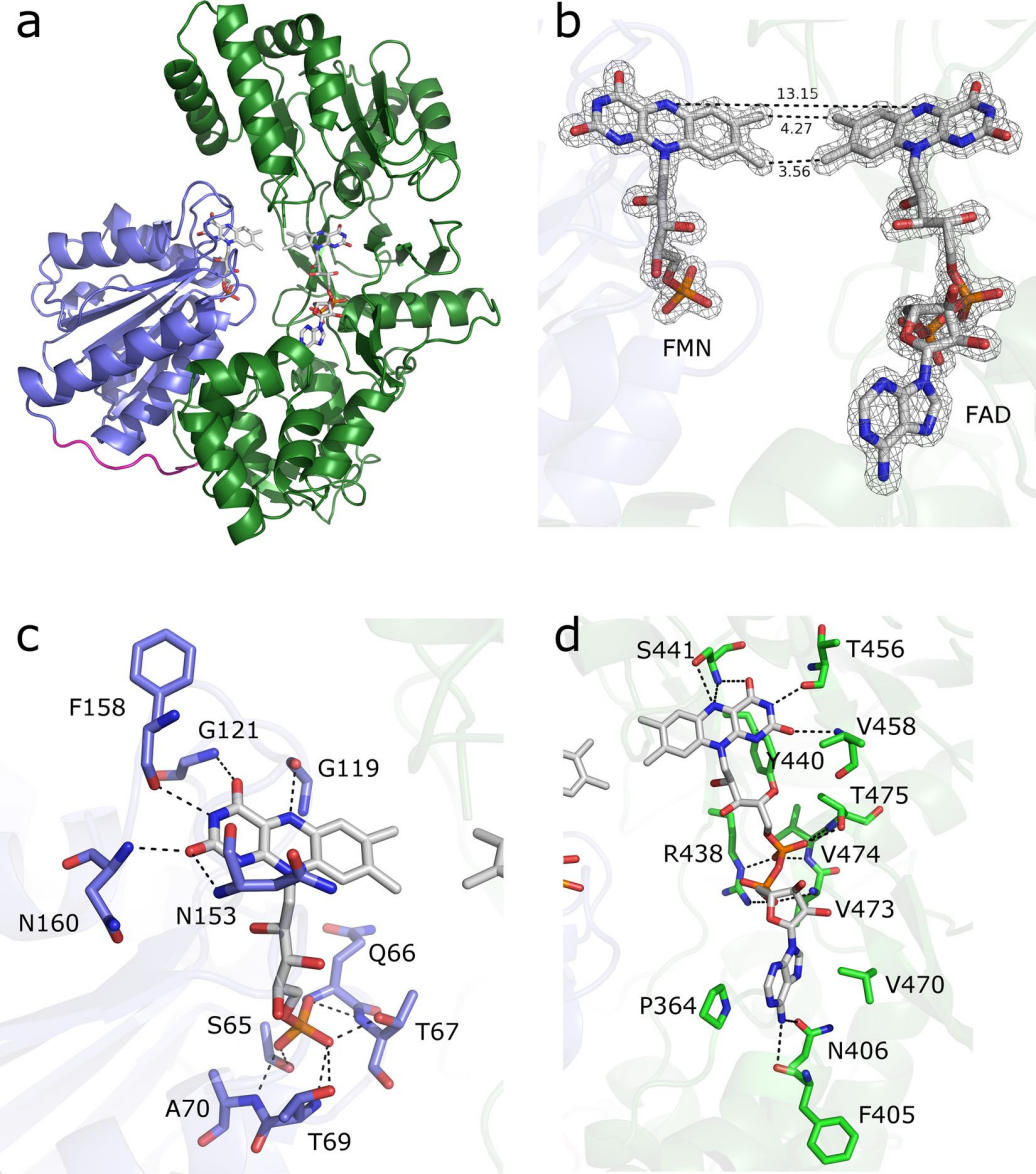
Crystal structure of *Ct*CPR. (**a**) Ribbon diagram showing the overall fold of *Ct*CPR, with cofactors modelled as ball and sticks. FMN-binding domain (blue), hinge loop (magenta) and FAD- and NADPH-binding domain (green). (**b**) FMN and FAD cofactors of *Ct*CPR with 2Fo-Fc electron density maps contoured at 2 σ. Interatomic distances depicted as dashed lines with distances in Å (**c**) FMN-binding and (**d**) FAD-binding in the active site of *Ct*CPR.

The X-ray structures of *Ct*CPR were solved with and without the cofactor, NADPH, at resolutions of 1.5 Å and 2.1 Å, respectively (Table S1). Both structures were obtained in the closed conformation (Fig. 3a) wherein the FMN-binding, flavodoxin-like domain interacts with the FAD and NADP- binding, ferredoxin-NADP^+^ reductase (FNR) like domain. In this conformation, the FMN and FAD moieties are in close proximity (~4.3 Å between the 7-methyl groups and ~3.6 Å between the 8-methyl groups, Fig. 3b), allowing direct electron transfer between the flavins. Thus far, most CPR structures have been solved in the closed conformation, including rat (rCPR)^21^, human (hCPR)^22^, and yeast (*Saccharomyces cerevisiae*, yCPR) CPRs^23^. The open conformation has only been obtained for a variant of hCPR with a 4-amino-acid deletion (ΔTGEE) in the hinge region^4^ and an N-terminally truncated yeast-human chimeric version of the enzyme^6^. More recently, a semi-open structure has been solved for the CPR from *Arabidopsis thaliana*^49^.

Even though only 22 residues were truncated at the N-terminus, density was only observed from residue 45, suggesting a relatively long flexible *N*-terminal region. The overall structure of *Ct*CPR is very similar to that of yCPR, rCPR and hCPR. Despite only sharing ~40% sequence identity with hCPR and rCPR, structural alignments revealed rmsd values of 1.5–1.6 Å, whereas yCPR (54% sequence identity) aligned with an rmsd of 1.1 Å.

Electron densities for both the FMN and FAD were well defined (Fig. 3b). The FMN-binding topology is conserved among the different CPRs, with the isoalloxazine ring hydrogen-bonded to backbone atoms in addition to some water-mediated hydrogen bonds. The phosphate group is similarly bound but also contacts directly to the sidechains of Ser65, Thr67 and Thr69 in addition to a more extensive water bridge network (Figs. 3c and S7). The FAD is bound in a fashion similar to that of yCPR where, in contrast to that observed for rCPR and hCPR (Fig. S9), the adenosine moiety is buried, sandwiched between Pro364 and Val470, and hydrogen-bonded via N6A to the side chain oxygen of Asn406 and the main chain carbonyl oxygen of Phe405 (Fig. 3d), in addition to various water-mediated contacts to other main chain atoms (Fig. S8). The Arg438 electrostatically stabilizing the FAD through interaction with the diphosphate is also conserved. Apart from the direct hydrogen bond formed with the side chain of Ser441, the isoalloxazine ring of FAD is mostly hydrogen-bonded to main-chain carbonyl and amine groups (Fig. 3d).

**Figure 4:**
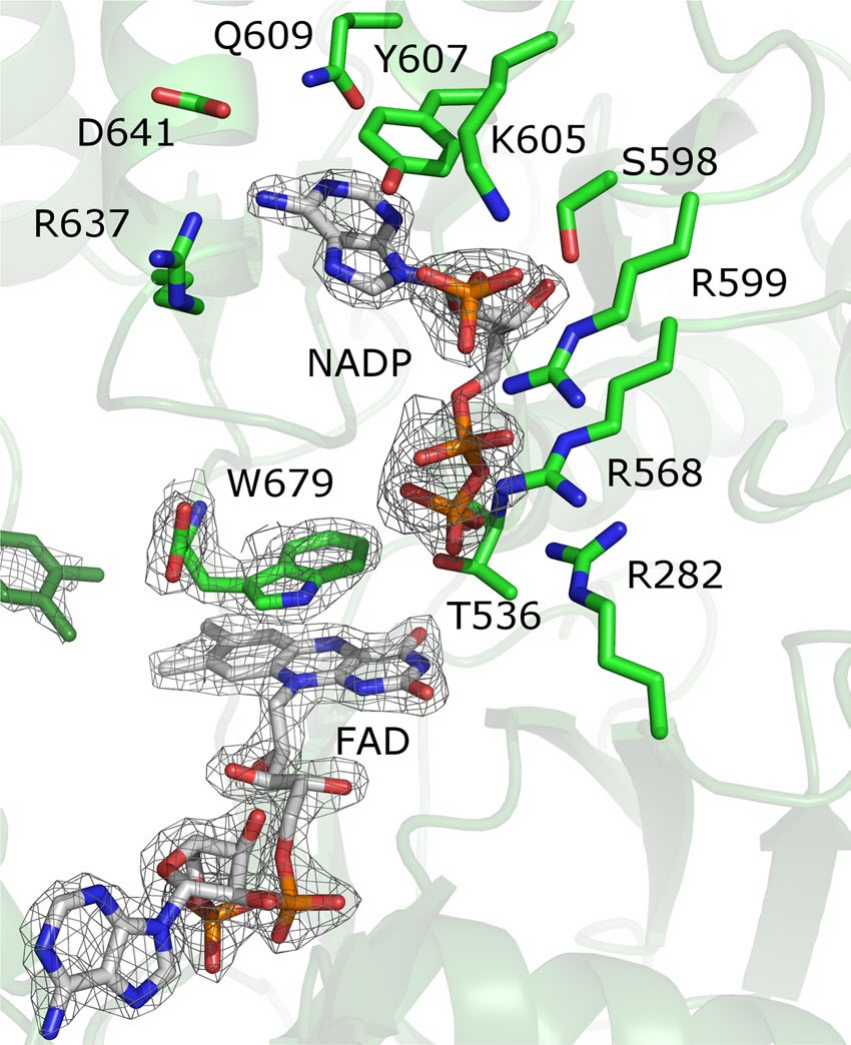
NADPH binding to *Ct*CPR. Residues interacting with the 3′-phosphate-adenosine-5′-diphosphate part of NADPH are shown. FAD and *C*-terminal Trp679 are also displayed. FAD and NADPH cofactors and terminal Trp of *Ct*CPR with 2Fo-Fc electron density maps contoured at 2 σ.

Despite the fact that *Ct*CPR crystallized in the presence of excess NADPH, the open structure was not observed. No differences were detected between the two structures, with the exception of the presence of the 3′-phosphate-adenosine-5′-diphosphate part of NADPH, which, in contrast to the FMN and FAD cofactors, is bound through mostly side chain interactions (Fig. 4). No density was observed for the nicotinamide mononucleotide moiety of the NAPDH, with the *C*-terminal Trp679 instead stacked on the *re*-side of the FAD. This residue is conserved among CPRs and has been implicated in the regulation of electron transfer^50,51^. rCPR and hCPR have extended C-termini, with the equivalent tryptophan as the penultimate residue. As the tryptophan sterically prevents the binding of the nicotinamide part of NADPH, movement is required for hydride transfer to occur. This process is coupled to the movement of the Asp634 loop, which is induced by the binding of NADPH^50^.

**Figure 5:**
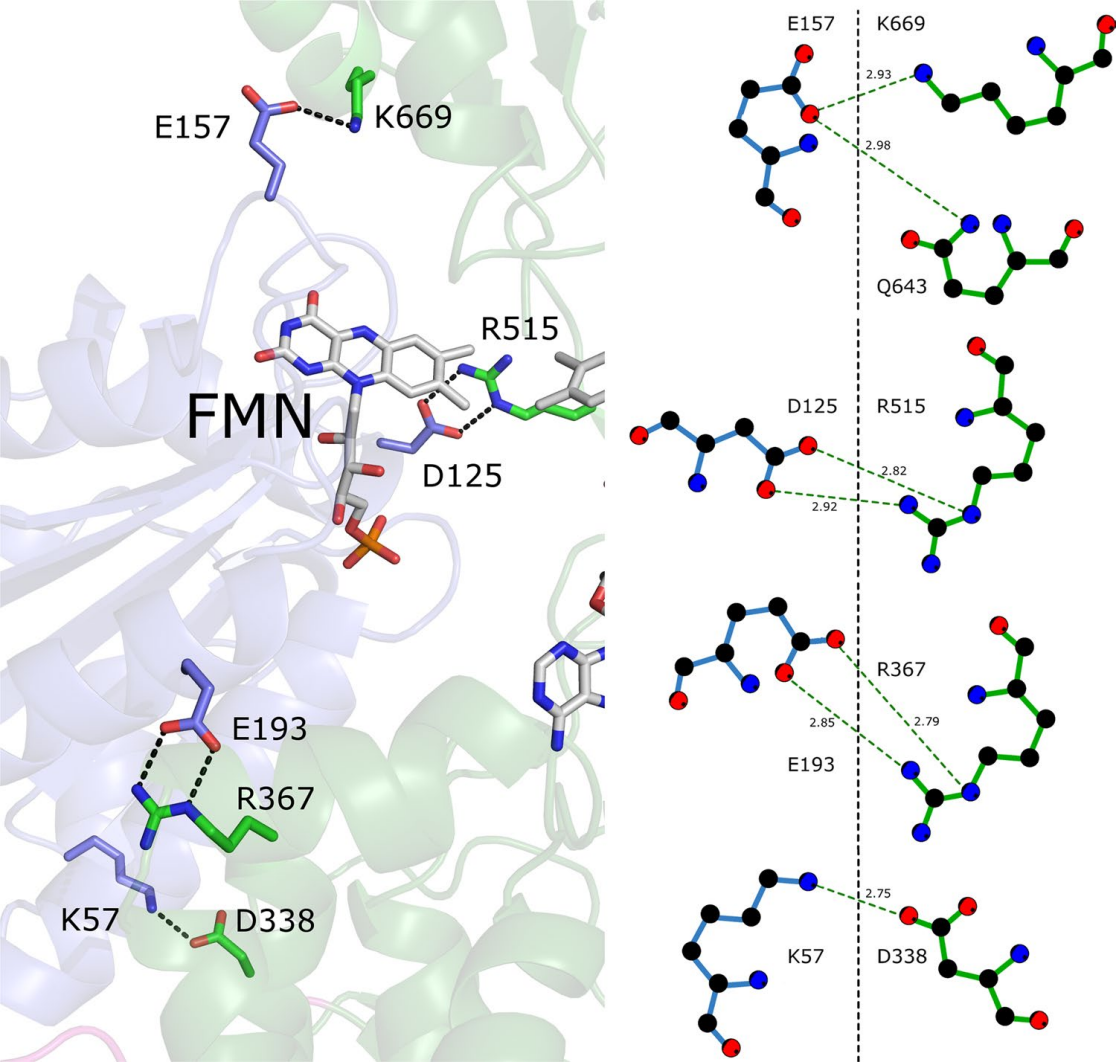
Salt bridges between residues in the interfaces of the FMN and FAD domains of *Ct*CPR.

The interface of the FMN and FAD-binding domains are stabilized with various salt-bridges and extensive hydrogen bonding networks (Fig. 5), suggesting strong interactions between the domains. These ionic interactions (or locational equivalents in other solved CPR structures) are mostly solvent-exposed and highly conserved among the different CPRs. These interactions will, however, be significantly weakened with increased salt concentrations such as that found under physiological conditions. The rate-limiting step in rCPR, hCPR and yCPR has been found to be the hydride transfer from NADPH to FAD, while interflavin electron transfer is the rate-limiting step in plant CPRs^31,32^. The transition between the open and closed formations could limit hydride transfer from NADPH to FAD, as well as interflavin electron transfer, as the resting state of CPR appears to be the closed conformation during which NADPH binding occurs. The two domains are linked via a flexible hinge region, allowing the FMN domain to rotate away from the FAD domain for electron transfer to its physiological partner proteins. This conformational freedom has to be countered so that once the enzyme returns to the closed conformation, the correct interfacial domain interaction is achieved. The conserved salt-bridges could possibly direct the domain associations for successful and productive orientations by reducing the potential relative orientations. Another important factor in the optimization of the open/closed movement is the flexibility of the hinge region. An example of this can be found in isoforms CPR1 and CPR2 from *Andrographis paniculate* (ApaCPR1 and ApaCPR2). The hinge of ApaCPR1 contains two Pro residues, while ApaCPR2 does not have any (Fig. S10). Kinetic parameters show that ApaCPR1 has a greater *k*_cat_ but a higher *K*_M_ than ApaCPR2 (Table 1) indicating a compromise between flexibility and activity. It has also been shown that the hinge is important for the conformational equilibrium in hCPR and therefore the reaction rate of the enzyme^52^. This region is highly conserved between rat and human CPRs, with some of the residues forming salt-bridges with the linker/FAD domain (Fig. S10). However, comparison of the amino acid sequences from CPRs from different organisms revealed that the hinge differs significantly in CPRs from different species and that these specific ionic interactions are not conserved. In yeast and *Ct*CPR, the hinge interacts mainly with the FMN domain (Fig S10). The differences in salt bridges formed in mammalian and yeast enzymes might influence the ability of the yeast-human chimeric protein to stabilize the closed conformation, promoting the open conformation which would have an effect on its activity^53^. These results suggest the importance of the ionic interactions in constraining the flexibility of the CPR to optimize proper electron transfer.

## Concluding Remarks

In this study, we describe the functional expression of the CPR from *C. tropicalis* in *E. coli* and the X-ray crystal structures of the closed conformation of the *Ct*CPR as well as the *Ct*CPR in complex with its natural cofactor, NADPH. Our findings highlight the major differences between mammalian and yeast/fungal CPRs, most notably the different binding orientation of the FAD. Molecular insights gained into the interfacial domain interactions suggest that specific conserved ionic interactions contribute to the flexibility and conformational equilibrium of the protein, which in turn, would affect reaction rates. Additionally, the high specific activity of *Ct*CPR makes it a promising redox partner for CYP-based biocatalytic reactions.

## Materials and Methods

### Cloning of the enzymes

The *cpr-b* gene from *C. tropicalis* 1230 (AAV84084.1) was synthesized by Epoch Life Sciences (USA). The sequence was optimized for *E. coli* expression and lacks the sequence coding for the *N*-terminal anchor (amino acids 2–22). Membrane-spanning regions of CPR were delineated using MEMSAT3 (http://bioinf.cs.ucl.ac.uk/psipred/). The *cpr-b* open reading frame was subcloned into pETDuet-1 using the *Nco*I/*Bam*HI sites located in the second multiple cloning site of the vector. The open reading frame of *cyp52a21* from *C. albicans* was amplified from genomic DNA and sub-cloned into the pCWori vector as described previously^43^. *Bacillus megaterium* glucose dehydrogenase (*Bm*GDH) was expressed from pET28b(+)^54^.

### Expression and purification

*E. coli* BL21-Gold (DE3) (Stratagene) was transformed with the construct and positive colonies were selected in LB-agar plates supplemented with 100 µg/mL ampicillin.

Cells were grown in ZYP-5052 auto-induction medium^55^, containing antibiotic, for 36 h at 28 °C. For expression of CYP52A21, media was supplemented with 0.5 mM δ-aminolevulinic acid hydrochloride and 50 μM FeCl_3_·6H_2_O. Cells were harvested by centrifugation (7000 × *g*, 10 min, 4 °C) and resuspended in buffer A (25 mM Tris-HCl, pH 8.0). Disruption of the cells was carried out by single passage through the One-Shot Cell disrupter System (Constant Systems Ltd) at 30 kpsi, followed by centrifugation (30 000 × *g*, 30 min, 4 °C) and ultracentrifugation (100 000 × *g*, 90 min).

Protein purification was a three-step procedure carried out using an ÄKTA pure automated system (GE Healthcare Life Sciences, USA). First, the soluble fraction containing *Ct*CPR was loaded onto a 5 mL HiTrap Q-FF column (GE Healthcare), previously equilibrated with buffer A. Protein was eluted using a linear gradient of increasing NaCl (0–500 mM). Fractions containing *Ct*CPR were pooled, concentrated through ultrafiltration (Amicon Ultra-30 kDa MWCO (Merck, USA)), and desalted by size-exclusion chromatography (SEC) using PD-10 columns (GE Healthcare, USA) equilibrated with buffer A. The desalted protein was subjected to a second round of anion exchange chromatography. The sample was loaded onto a 5 mL HiTrap Q HP column (GE Healthcare) and the same purification protocol was applied. Finally, size-exclusion chromatography was performed using Sephacryl S200HR (XK 26 column, GE Healthcare) equilibrated with buffer A. Eluted *Ct*CPR was pooled, concentrated and stored at 4 °C for up to 15 days. For purification of CYP52A21 and *Bm*GDH, the soluble fractions were loaded onto a HisTrap FF column, equilibrated with buffer B (25 mM Tris-HCl pH 8.0; 40 mM imidazole, 500 mM NaCl), and the protein eluted with a linear gradient of imidazole (40–500 mM). The last step consisted of a SEC using buffer A (containing 10% (w/v) glycerol for CYP52A21). The eluted enzyme was pooled and concentrated as described above.

### Protein determination

Protein concentration was determined by the BCA assay (Pierce, USA) using bovine serum albumin as a standard. The concentration of active *Ct*CPR was estimated by the average absorbance at 380 nm (ε = 16.1 mM^−1^ cm^−1^) and 453 nm (ε = 17.9 mM^−1^ cm^−1^)^56^. CYP52A21 concentration was determined using CO-difference spectra at 450 nm (ε = 91 mM^−1^ cm^−1^). Protein purifications were analysed by SDS-PAGE.

### *Ct*CPR activity and kinetic analysis

The activity was evaluated using cytochrome *c* (horse heart, Sigma-Aldrich, UK) and FeCN (Sigma-Aldrich) as electron acceptors. Assays were performed at 25 °C in a 1 cm path-length cuvette using DU800 spectrophotometer (Beckman Coulter, USA). Reduction of cytochrome *c* and FeCN were measured at 550 (ε = 21000 M^−1^ cm^−1^) and 420 nm (ε = 1040 M^−1^ cm^−1^), respectively. Reaction mixtures contained 25 mM Tris-HCl (pH 8), 1.5 µM *Ct*CPR, and varying concentrations of the corresponding acceptor. The reaction was initiated by the addition of 150 µM NADPH in a final volume of 1 mL. Kinetic constants were determined by fitting the data to the Michaelis-Menten equation using Origin software (OriginLab, USA).

### Titration of oxidized *Ct*CPR

*Ct*CPR (50 µM) was reduced with different concentrations of NADPH (0–500 µM). Reactions were performed at 20 °C in buffer A. UV-visible spectra were recorded before and after the addition of NADPH.

### Uncoupling assays

For uncoupling determination, hydrogen peroxide production was quantified using Ampiflu^TM^ Red assay^19^ in the presence and absence of cytochrome *c*. Reactions were carried out in a microtiter plate using the Spectramax M2 spectrophotometer that allowed to follow simultaneously the consumption of NADPH (340 nm), reduction of cytochrome *c* (550 nm) and production of H_2_O_2_ (560 nm ε = 71000 M^−1^ cm^−1^).

The reaction was divided in two parts: A) Measurement of the consumption of NADPH and reduction of cytochrome *c*. Reactions were carried out in a total volume of 100 µL for 10 min, followed by inactivation of the enzyme at 80 °C. Unless otherwise specified, reactions contained 20 mM Tris-HCl pH 8.0, 0.5 µM *Ct*CPR and 0 or 25 µM cytochrome c. Reactions were started with the addition of 12.5 µM NADPH. B) Measurement of H_2_O_2_ production consisted on the addition of 100 µL Ampiflu solution (100 µM prepared in 200 mM sodium phosphate pH 8.0), 0.2 U/mL horseradish peroxidase (HRP) and 2 U/mL superoxide dismutase (SOD), and measurement of the reaction for 10 min. Reactions were performed at 20 °C, unless otherwise indicated.

### Crystallization, data collection and structure determination

The purified protein was concentrated to 6 and 8 mg/mL. Crystals were grown by sitting drop vapour diffusion method mixing 1 µL of *Ct*CPR and 1 µL of the reservoir solution (4% Tacsimate pH 5.0, 12% PEG 3350). For crystals with NADPH, the enzyme was crystallized in 5 x molar excess of the cofactor (0.2 M Sodium malonate pH 5.0, 20% PEG3350). Plates were incubated at 16 °C. Crystals were soaked in reservoir solution containing 30% (v/v) glycerol prior to cryocooling. X-ray diffraction data were collected at Diamond Synchrotron (UK) on beamline i04-1 (0.91587 Å) at 93 K. Data were processed using autoPROC, with indexing and integration using XDS^57^ and POINTLESS^58^, with intensities scaled and merged using AIMLESS^59^. Molecular replacement was performed using PHASER^60^ with yCPR (PDB:2BN4) as a search model. Iterative cycles of manual model building in COOT^61^ and refinement using refmac^62^ were performed and model building progress was monitored by changes in R_free_ and R_work_ values after each refinement cycle.

Coordinates and structure factors have been deposited in the Protein Data Bank (PDB) under the codes 6T1U (*Ct*CPR) and 6T1T (*Ct*CPR in complex with NADPH).

## Supplementary information

Supplementary information
