# Supplementary material for: Biochemical and structural insights into the cytochrome P450 reductase from Candida tropicalis
Source: Sci Rep. 2019 Dec 27;9:20088. doi: 10.1038/s41598-019-56516-6 (PMC6934812; doi:10.1038/s41598-019-56516-6)
Supplement: Supplementary file 1 — Supplementary information [file 41598_2019_56516_MOESM1_ESM.pdf]

Biochemical and Structural Insights into the Cytochrome P450 reductase from *Candida tropicalis*

Ana C. Ebrecht[1,3], Naadia van der Bergh[2,3], Susan T. L. Harrison[2,3], Martha S. Smit[1,3], B. Trevor Sewell[4*], Diederik J. Opperman[1,3*]

[1]Department of Microbial, Biochemical, and Food Biotechnology, University of the Free State, Bloemfontein, 9301, South Africa

[2]Centre for Bioprocess Engineering Research (CeBER), Department of Chemical Engineering, University of Cape Town, Rondebosch, Cape Town 7701, South Africa

[3]South African DST-NRF Centre of Excellence in Catalysis (c*Change), University of Cape Town, Private Bag, Rondebosch, Cape Town 7701, South Africa

[4]Structural Biology Research Unit, Department of Integrative Biomedical Sciences, Institute for Infectious Diseases and Molecular Medicine, University of Cape Town, Cape Town, 7700, South Africa

*trevor.sewell@uct.ac.za

*opperdj@ufs.ac.za

Supplementary Information

Figure S1: Multiple sequence alignment of CPRs of different species.

Figure S2: Saturation curves of *Ct*CPR for (A) ferricyanide and (B) cytochrome *c.*

Figure S3: GC-MS chromatograms and mass spectra of biotransformations of dodecanoic acid by CYP52A21.

Figure S4: Control of the consumption of NADPH with the different components of the reaction.

Figure S5: Control reactions of the quantification of H_2O_2 stability.

Figure S6: Control reactions of the total H_2O_2 detected with and without SOD.

Table S1. Data collection and refinement statistics. Values in parentheses are for highest resolution shell.

Figure S7: Interactions of the FMN group of *Ct*CPR with the residues and water molecules.

Figure S8: Interactions of the FAD group of *Ct*CPR with the residues and water molecules.

Figure S9: Alignment between the FMN and FAD groups of the different CPR structures.

Figure S10: Interaction of the hinge region with the FMN and FAD domains for CPRs from different species.

Table S2: Uncoupling percentage (%) relative to NADPH consumed at different ionic strength.

Table S3: Uncoupling percentage (%) relative to NADPH consumed at different pH.

Table S4: Uncoupling percentage (%) relative to NADPH consumed at different temperatures.

Figure S11: Full-length SDS-PAGE gel used in the preparation of the cropped insert for Figure 1 in the main text.

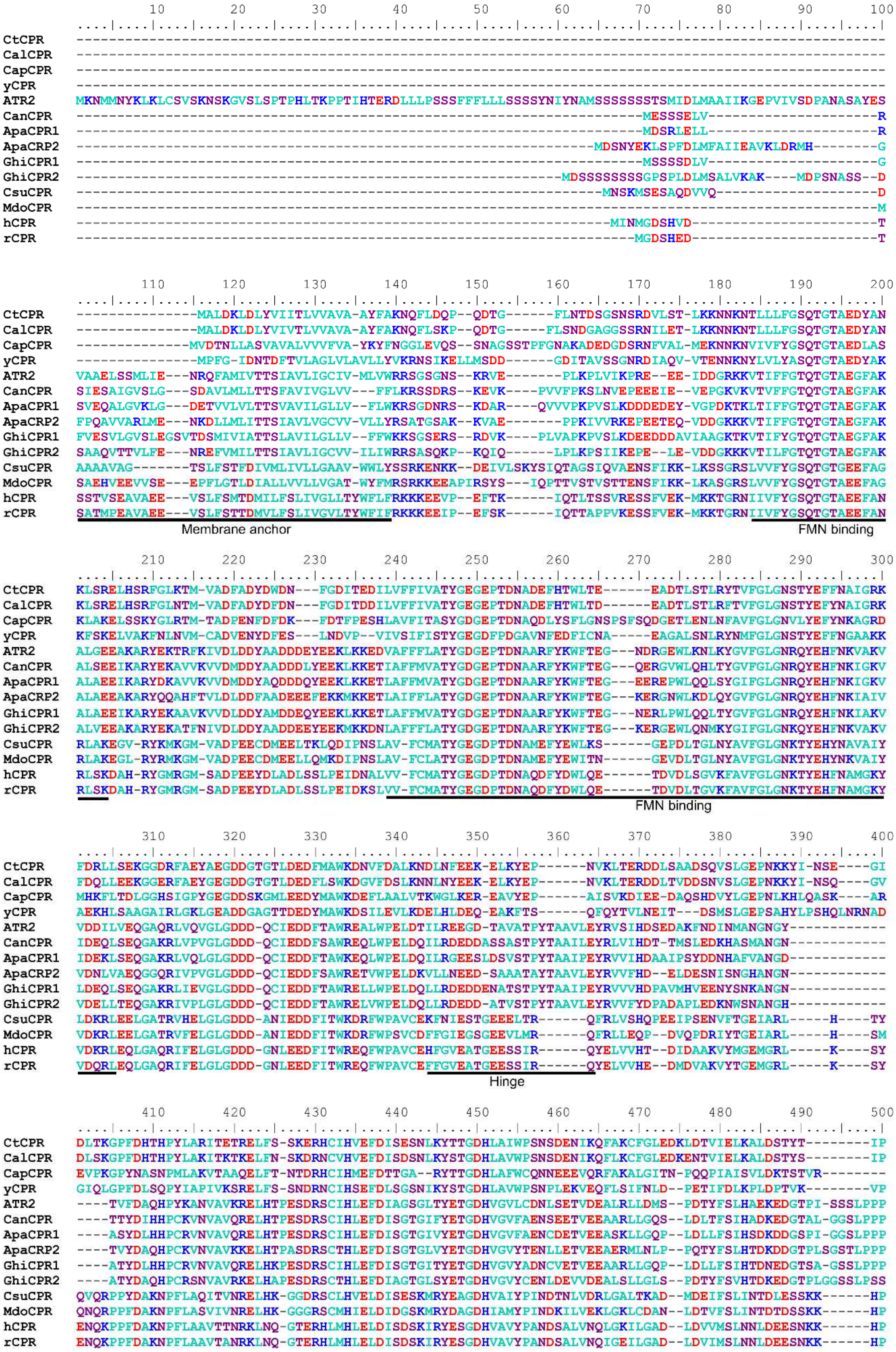
CtCPR
CalCPR
CapCPR
yCPR
ATR2
CanCPR
ApaCPR1
ApaCRP2
GhiCPR1
GhiCPR2
CsuCPR
MdoCPR
hCPR
rCPR
10 20 30 40 50 60 70 80 90 100
110 120 130 140 150 160 170 180 190 200
Membrane anchor
FMN binding
210 220 230 240 250 260 270 280 290 300
FMN binding
310 320 330 340 350 360 370 380 390 400
Hinge
410 420 430 440 450 460 470 480 490 500

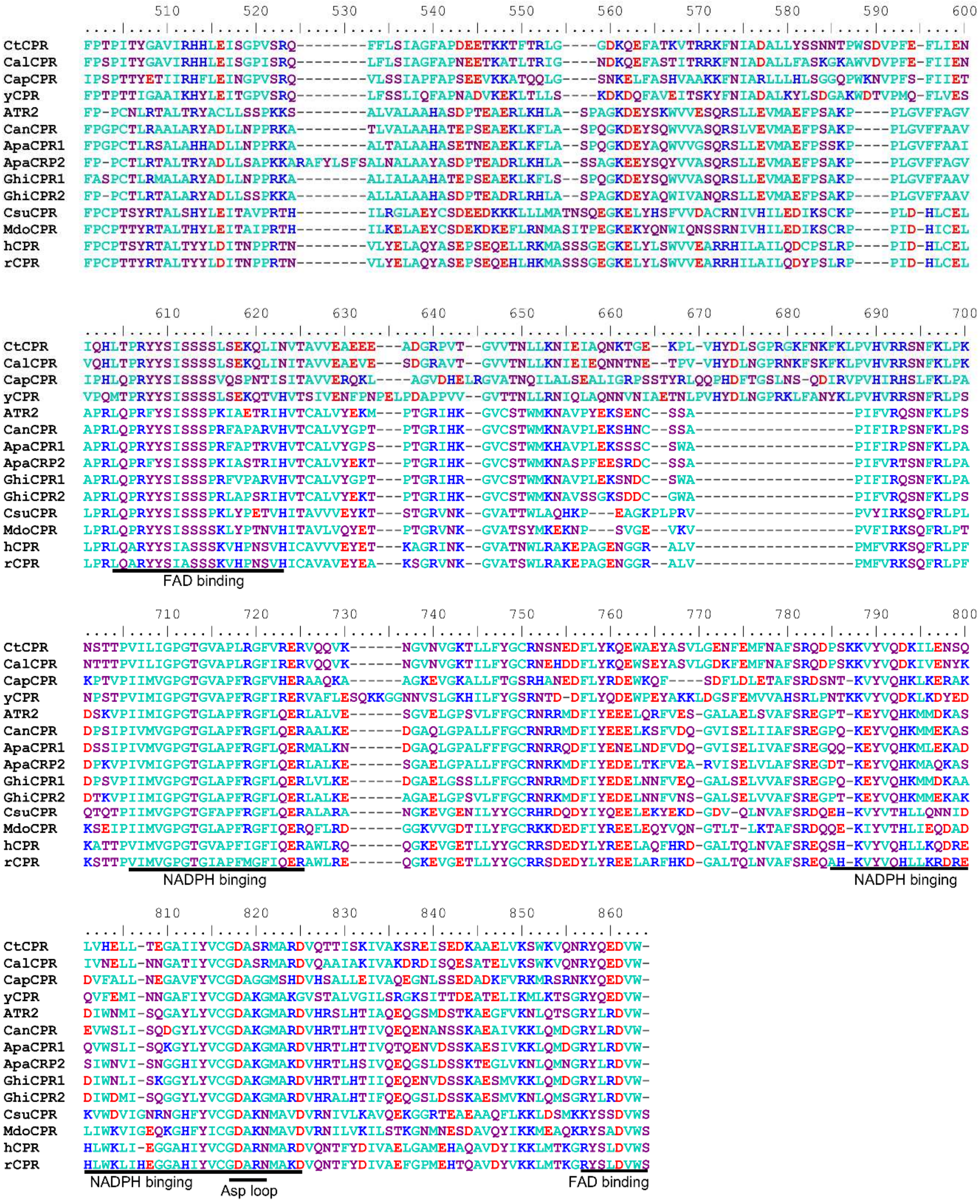

Figure S1. Multiple sequence alignment of CPRs of different species generated by ClustalW. CtCPR, *Candida tropicalis* (AAV84084.1); CalCPR, *Candida albicans* (KGQ86567); CapCPR, *Candida apicola* (JQ015264); Ycpr, *Saccharomyces cerevisiae* (P16603); ATR2, *Arabidopsis thaliana* CPR2 (AEE85738.1); CanCPR, *Capsicum annuum* (AFV95075); ApaCRP1, *Andrographis paniculate* CPR1 (AQT38168); ApaCRP2, *A. paniculate* CPR2 (AQT38169); GhiCPR1, *Gossypium hirsutum* CPR1 (ACN54323); GhiCPR2, *G. hirsutum* CPR2 (ACN54324); CsuCPR, *Chilo suppressalis* (AGM20565); MdoCPR, *Musca domestica* (Q07994); hCPR, human (NP_000932); rCPR, rat (NP_113764).

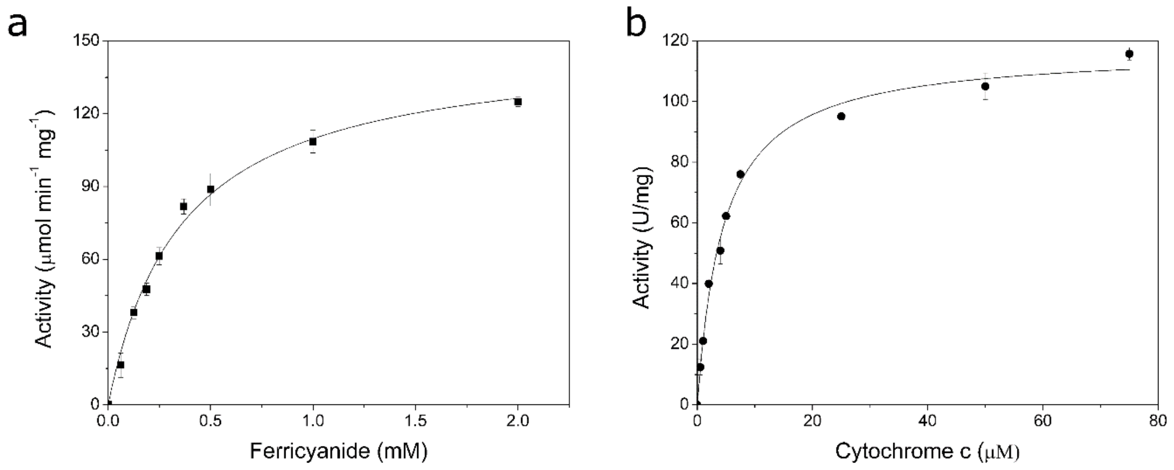

Figure S2. Saturation curves of *Ct*CPR for (**a**) ferricyanide and (**b**) cytochrome *c*. Values are the average of three independent measurements. Data was fit into the Michaelis-Menten equation.

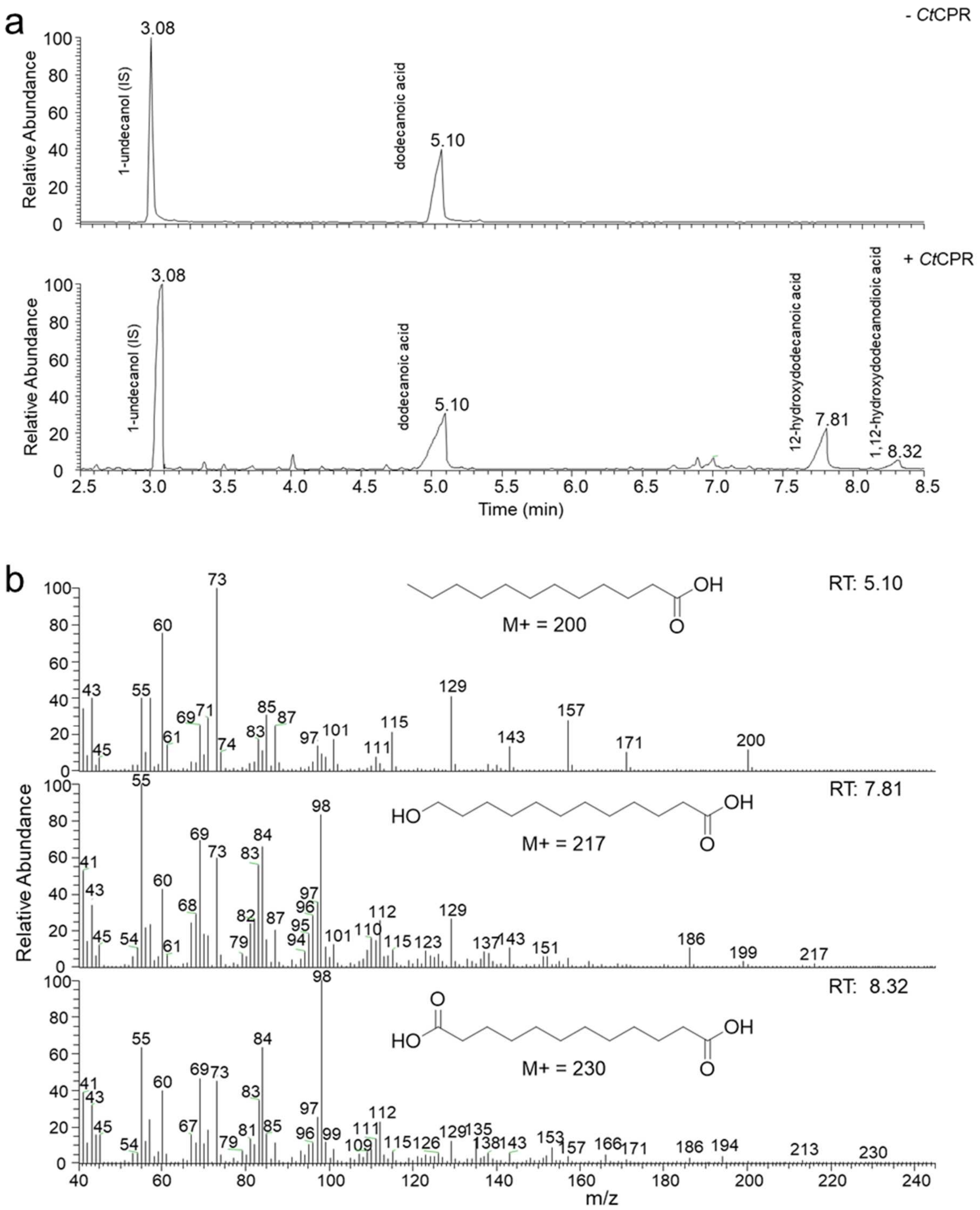

Figure S3. GC-MS chromatograms and mass spectra of biotransformations of dodecanoic acid by CYP52A21. (**a**) Chromatograms of the biotransformations without (- *Ct*CPR) and with (+ *Ct*CPR) addition of *Ct*CPR. (**b**) Mass spectra of substrate dodecanoic acid (RT: 5.10), and products 12-hydroxydodecanoic acid (RT: 7.81), and 1,12-dodecanedioic acid (RT:8.32). Reaction conditions: buffer = 200 mM potassium phosphate pH 8.0, [CYP52A21] = 2 µM, [*Ct*CPR] = 50 µM, [dodecanoic acid] = 4 mM, [glucose] = 200 mM, [glycerol] = 100 mM, [1,2-diodecanoyl-*sn*-glycero-3-phosphocoline] = 45 µM, [*Bm*GDH] = 0.2 U, [$NADP^+$] = 0.5 mM. Reactions were incubated at 30°C for 24 hours with shaking (200 rpm).

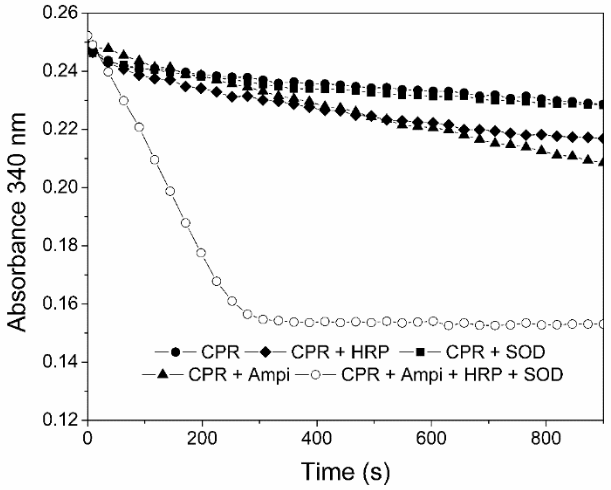

Figure S4. Control of the consumption of NADPH with the different components of the reaction. The assays were carried in a final volume of 200 µL, containing 20 mM Tris-HCl pH 8.0, 0.2 µM *Ct*CPR and started with the addition of 12.5 mM NADPH (black circle). The different reactions contained: 2 U/mL superoxide dismutase (SOD, square), 0.2 U/mL horseradish peroxidase (HRP, diamond), 50 µM Ampiflu^{TM} Red (Ampi, triangle) and HRP, SOD and Ampiflu^{TM} Red (white circle).

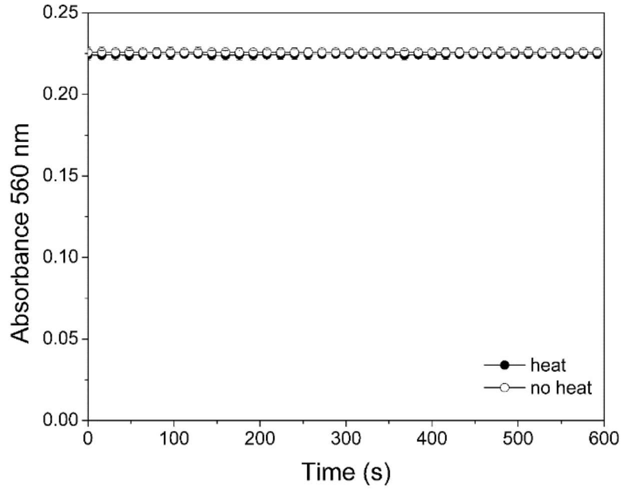

Figure S5. Control reactions of the quantification of H_2O_2 stability. Ampiflu^{TM} Red/HRP reaction was incubated with 8 mM H_2O_2, one sample was heated at 80 °C (black) for 1 min and, after cooling down, the absorbance was measured and compared to reaction without the heating step (white).

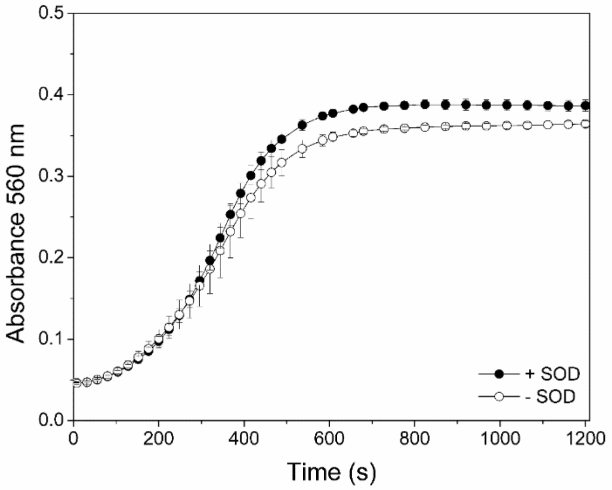

Figure S6. Control reactions of the total H_2O_2 detected during the reactions with (black) and without (white) addition of SOD. Plot shows increase on absorbance at 560 nm over time.

	*Ct*CPR	*Ct*CPR with NADPH
**Data collection**		
Space group	P 1 21 1	P 1 21 1
Cell dimensions		
a, *b*, *c* (Å)	74.29, 67.18, 143.47	74.66, 67.50, 143.99
α, β, γ (°)	90.00, 91.50, 90.00	90.00, 91.55, 90.0
Resolution (Å)	52.27 - 1.50 (1.55 - 1.50)*	74.63 - 2.08 (2.16 - 2.08)
R_{merge}	0.081 (0.687)	0.191 (2.247)
<*I* /σ*I*>	7.7 (1.5)	4.0 (0.5)
Completeness (%)	97.5 (96.8)	95.4 (86.9)
Redundancy	3.4 (3.5)	3.0 (2.9)
$CC_{1/2}$	0.99 (0.72)	0.99 (0.36)
**Refinement**		
Resolution (Å)	52.27 - 1.50	74.63 - 2.08
No. reflections used	220 256	82 237
R_{work} / R_{free}	0.176/0.202	0.219/0.264
Molecules in ASU	2	2
No. atoms		
Protein	10 253	10 184
Ligand/ion	62 (FMN), 106 (FAD)	62 (FMN), 106 (FAD), 62 (NADPH)
Water	964	371
B-factors		
Protein	23.0	41.7
Ligand/ion	16.2 (FMN), 14.7 (FAD)	41.5 (FMN), 28.7 (FAD), 36.4 (NADPH)
Water	30.1	38.9
R.m.s. deviations		
Bond lengths (Å)	0.014	0.007
Bond angles (°)	1.683	1.474
Ramachandran distribution (%) Favoured/Allowed/Outliers	98.0/1.9/0.2	95.7/3.9/0.4

Table S1. Data collection and refinement statistics. Values in parentheses are for highest resolution shell.

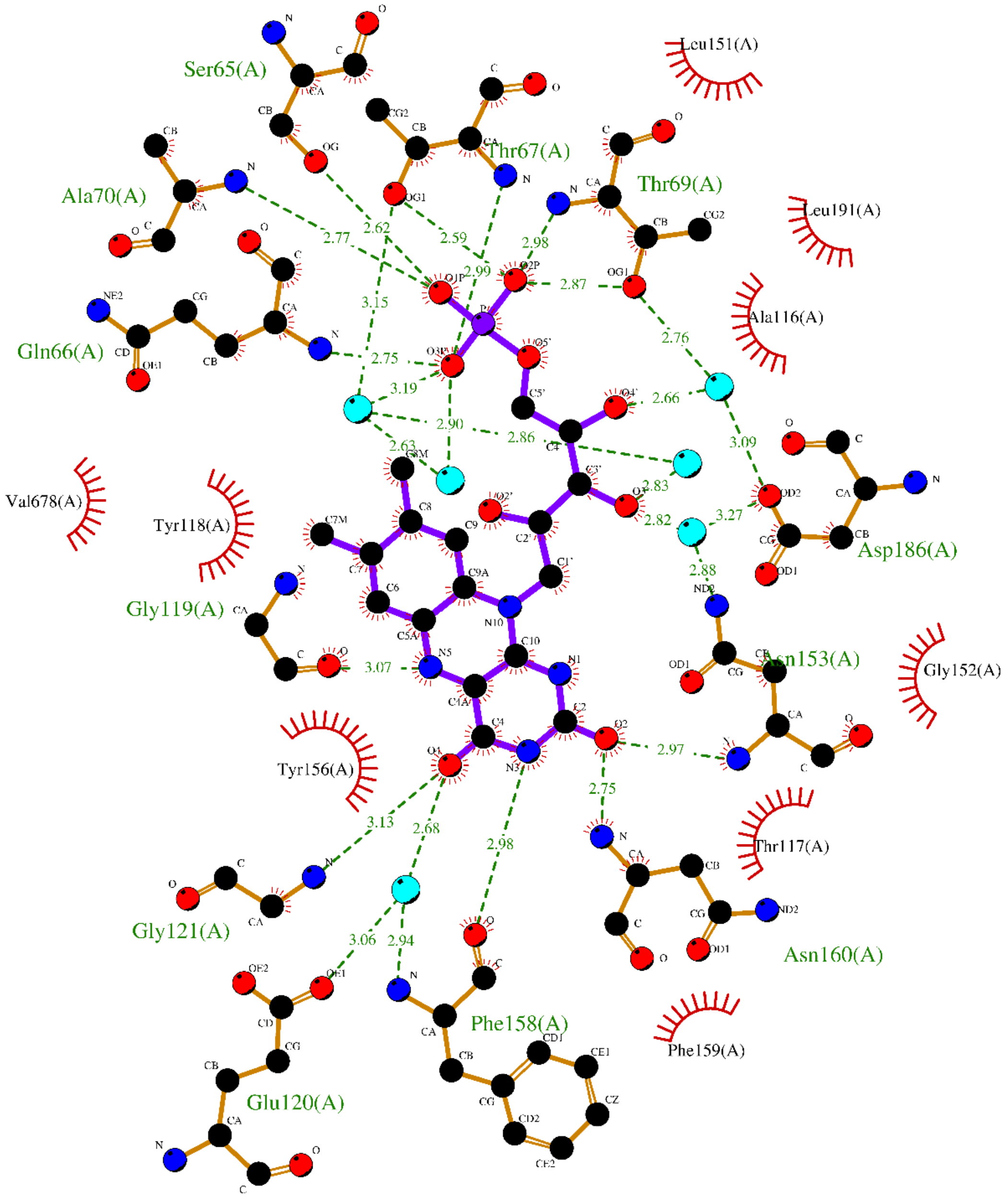

Figure S7. Interactions of the FMN group of *Ct*CPR with the residues and water molecules.

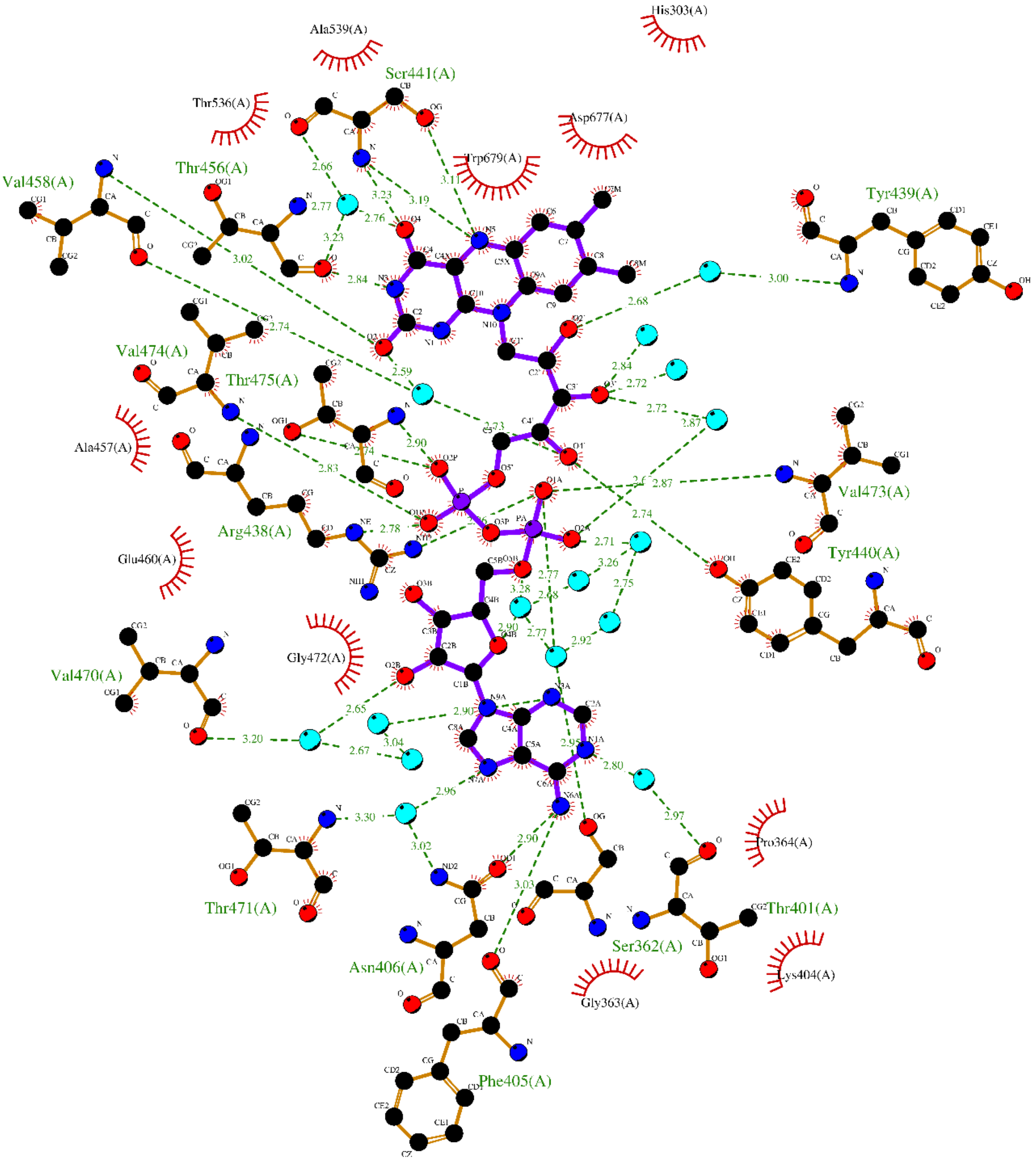

Figure S8. Interactions of the FAD group of *Ct*CPR with the residues and water molecules.

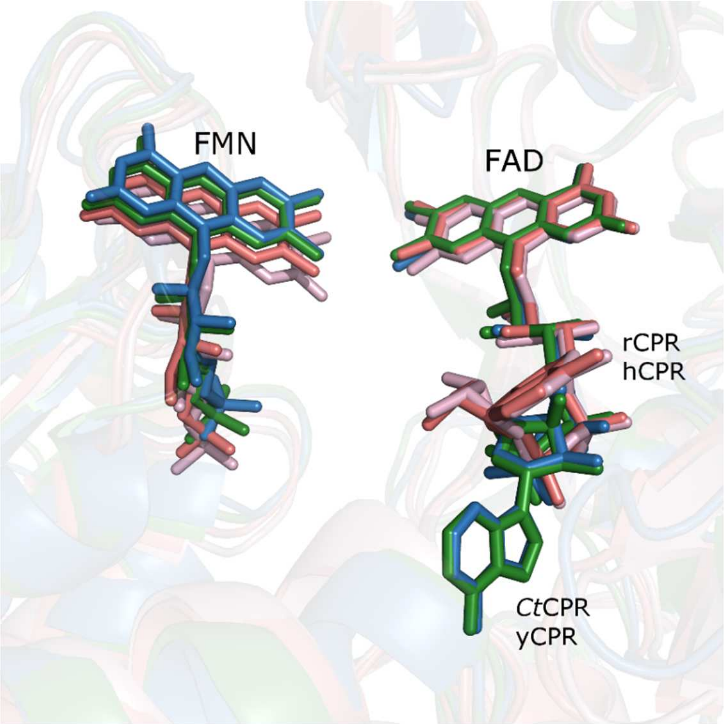

Figure S9. Alignment between the FMN and FAD groups of the different CPR structures. *Ct*CPR (green) CPR from *C. tropicalis.* yCRP (blue) *S. cerevisiae* CPR. hCPR (pink) human CPR. rCPR (salmon) rat CPR.

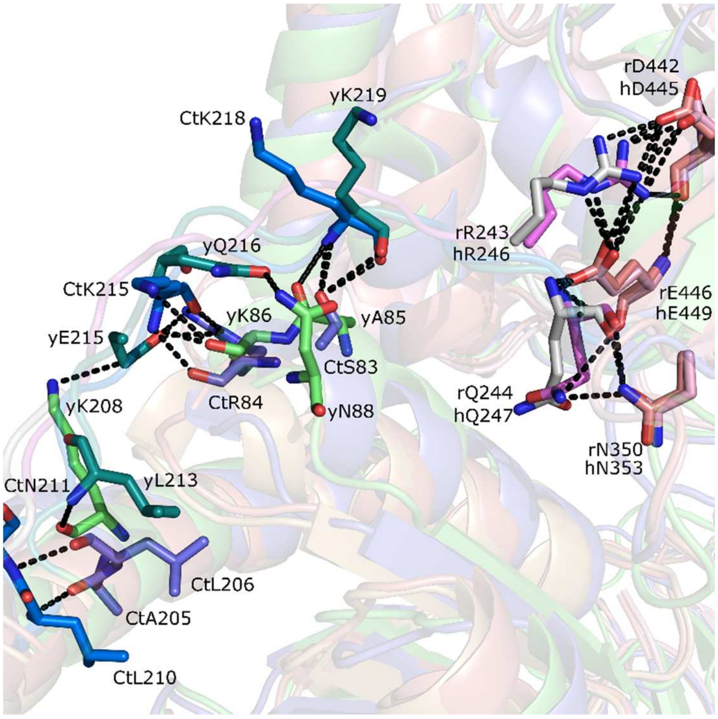

Figure S10. Interaction of the hinge region with the FMN and FAD domains for CPRs from different species. *C. tropicalis* (Ct), yeast *S. cerevisiae* (y), rat (r) and human (h) CPR structures are aligned. Residues from the hinge are displayed Ct (blue), y (green), r (grey), h (magenta) and their interactions with amino acids from the FMN domain (light blue for Ct and light green for y) and the FAD domain (salmon for r and pink for h).

NaCl (M)						
	0	0.1	0.15	0.2	0.5	1
NADPH	100	100	100	100	100	100
H_2O_2	27	33	33	31	36	51
Cytochrome c	73	67	67	69	64	49

Table S2. Uncoupling percentage (%) relative to NADPH consumed at different ionic strengths.

pH						
	5.5	6.0	6.5	7.0	7.5	8.0
NADPH	100	100	100	100	100	100
H_2O_2	34	30	27	28	29	28
Cytochrome c	66	70	73	72	71	72

Table S3. Uncoupling percentage (%) relative to NADPH consumed at different pH.

T (°C)				
	20	25	37	42
NADPH	100	100	100	100
H_2O_2	28	31	24	29
Cytochrome c	72	69	76	71

Table S4. Uncoupling percentage (%) relative to NADPH consumed at different temperatures.

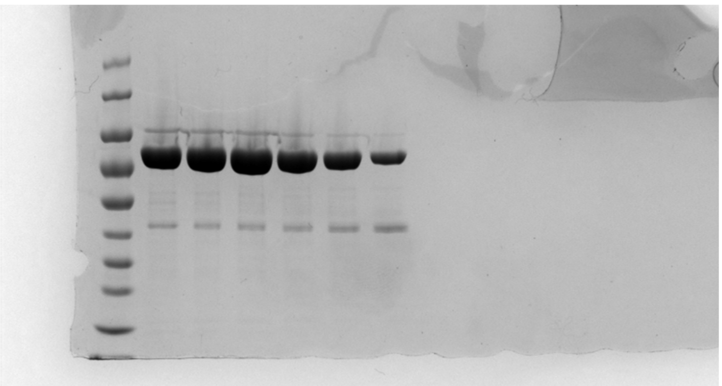

Figure S11: Full-length SDS-PAGE gel used in the preparation of the cropped insert for Figure 1 in the main text. Lane 1 represent the molecular weight marker, with subsequent lanes the eluted fractions from gel filtration chromatography.
